# Gene differential coexpression analysis based on biweight correlation and maximum clique

**DOI:** 10.1186/1471-2105-15-S15-S3

**Published:** 2014-12-03

**Authors:** Chun-Hou Zheng, Lin Yuan, Wen Sha, Zhan-Li Sun

**Affiliations:** 1 College of Electrical Engineering and Automation, Anhui University, Hefei 230601, China; 2 College of Information and Communication Technology, Qufu Normal University, Rizhao 276800, China

## Abstract

Differential coexpression analysis usually requires the definition of 'distance' or 'similarity' between measured datasets. Until now, the most common choice is Pearson correlation coefficient. However, Pearson correlation coefficient is sensitive to outliers. Biweight midcorrelation is considered to be a good alternative to Pearson correlation since it is more robust to outliers. In this paper, we introduce to use Biweight Midcorrelation to measure 'similarity' between gene expression profiles, and provide a new approach for gene differential coexpression analysis. Firstly, we calculate the biweight midcorrelation coefficients between all gene pairs. Then, we filter out non-informative correlation pairs using the 'half-thresholding' strategy and calculate the differential coexpression value of gene, The experimental results on simulated data show that the new approach performed better than three previously published differential coexpression analysis (DCEA) methods. Moreover, we use the maximum clique analysis to gene subset included genes identified by our approach and previously reported T2D-related genes, many additional discoveries can be found through our method.

## Background

DNA Microarray has been widely used as measurement tools in gene expression data analysis [1-4]. Gene expression profiling data from DNA microarray can detect the expression levels of thousands of genes simultaneously. Which provide an effective way for mining disease-related genes nalysis of gene expression data can be divided into three levels: firstly, analysis the expression level of individual genes, and to determine its function based on gene expression level changes under different experimental conditions. For example, the tumor type specific genes are identified according to the significance of difference in gene expression using the statistical hypothesis testing analysis method. Secondly, study gene interaction and co-regulation through the combination of genes and grouping. Finally, attempt to deduce the potential gene regulatory networks mechanism and explain the observed gene expression data.

Among the microarray data analysis methods, gene differential expression analysis is one of the most widely used types of analysis for disease research. Gene differential expression analysis method selects differentially expressed genes according to expression change value of a single gene. Gene expression value change between normal samples and disease samples can be used to present the possibility of the relation between gene and disease. However, the traditional pathogenicity genes selection methods based on gene expression data treats each gene individually and the interaction between them is not considered. Actually, genes and their protein products do not perform their functions in isolation [5,6], but in cooperation. Functional changes such as alteration in tumor cell growth process, energy metabolism and immune activity are accompanied with gene coexpression changes. Differentially expressed genes selection methods often focus only on the size of the single gene and disease relation, ignoring a plurality of pathogenic genes of the complex disease as a gene module with disease related, as well as within the module gene [7].

Differential coexpression analysis, as a more comprehensive technique to the differential expression analysis, was raised to research gene regulatory networks and biological pathways of phenotypic changes through measure gene correlation changes between disease and normal conditions. Differential coexpression genes are defined as genes whose correlated expression pattern differs between classes [8]. The gene coexpression changes between different conditions indicate gene regulatory pathways and networks associated with disease. In gene differential coexpression analysis, a pair of gene expression datasets under disease and normal conditions are transformed to a pair of coexpression matrix in which links represent transcriptionally correlated gene pairs, and then the differential coexpression score is calculated for each gene [9].

Until now, methods for differential coexpression analysis of gene expression data have been extensively researched, and multiple algorithms have been developed and tested [10-13]. Carter [10] mined the molecular characteristics of the cell state through gene coexpression topology method. Stuart et al. [14] and Bergmann et al. [15] separately constructed the gene coexpression network that connected genes whose expression profiles were similar across different organisms. They showed that functionally related genes are frequently coexpressed across organisms constituting conserved transcription modules [5]. Graeber [16] and Choi [5] both studied cancer from the perspective of differential coexpression. They found some genes were not be detected from the perspective of gene differential expression analysis. Butte [17] found gene coexpression modules based on a new gene expression similarity measure method, i.e., mutual information. Varadan [18] searched for disease-related gene differential coexpression modules from all gene subsets by entropy minimization and Boolean reduction methods (EMBP). Bansal [19], Della Gtta [20] and Lorenz [21] used linear regression method to excavate relation of gene transcription and regulation separately. In those gene differential coexpression analysis methods, the most common choice of similarity measurement is Pearson correlation coefficients [5,11,12,22]. However, Pearson correlation is sensitive to outliers. Biweight midcorrelation (bicor) is considered to be a good alternative to Pearson correlation since it is more robust to outliers [23].

Graph theoretical concepts are useful for description and analysis of interaction and relationships in biological systems. The maximum clique problem (MCP) is a classical combinatorial optimization problem in graph theory. In 1957, Harary and Ross first proposed the deterministic algorithm to solve the maximum clique problem [24]. Since then many researchers have presented a variety of algorithms to solve this problem. The maximum clique problem is widely used in different areas, such as signal transmission, computer vision, and biological research etc. In this paper, we will use the concept of maximum clique to further investigate the identified gene set to gain insight into coexpression relationship between genes. For the sake of convenience, we use the terms graph and network interchangeably, the former stressing the mathematical concept, the latter the application. A graph consists of a set of nodes and a set of edges that connect the nodes. For a graph G = (V, E), the graph G is specified by the set of nodes V which also means genes in gene coexpression network, and the set of edges E which also represents gene coexpression relationships in gene network. A maximum clique means a clique which is a subset of the nodes in V that every pair of nodes in the subset is joined by an edge and is not a proper subset of any other clique.

However, the requirement of complete connectivity for gene maximum clique is restrictive. For real biological data network, its data size is large and has very complex relationships between the network nodes. When dealing with imperfect systems or with experimental data, we may need to consider more general notions of cohesive subgroups [25]. Our description here follows that of [26], they consider different notions of cohesive subgroups that include *n*-clique, *k*-plexes. In the analysis of gene expression data, genes closely linked functional module is not the strict sense of maximum clique due to the lack of certain section. We use density to measure approximation degree of functional module with maximum clique, making it more biological significance. In our study, we used the maximum clique concept to mine disease-related differential coexpression gene cluster.

In this paper, we propose a new approach for gene differential coexpression analysis based on Biweight Midcorrelation and half-threshoding strategy. Biweight midcorrelation is used to measure coefficients between the all gene pairs in normal condition and disease condition separately. The two gene correlation datasets are encoded into a pair correlations matrix over all gene pairs. We then filter out non-informative correlation pairs using the half-thresholding strategy and calculate the differential coexpression value of gene. We apply the new approach to a simulate dataset and a pair of type 2 diabetes(T2D) in rats datasets. Moreover, the maximum clique analysis are used to analyse the gene subset identified by our new approach and previously reported T2D-related genes in the dataset. In the light of this observation, we are confident that our method has a high potential for generating relevant hypotheses in biological and clinical research.

## Methods

### Biweight midcorrelation

Differential coexpression analysis usually requires the definition of 'distance' or 'similarity' between measured datasets, and the most common choice is Pearson correlation coefficient. However, Pearson correlation coefficient is sensitive to outliers. Biweight midcorrelation is considered to be a good alternative to Pearson correlation since it is more robust to outliers [23].

**Figure 1:**
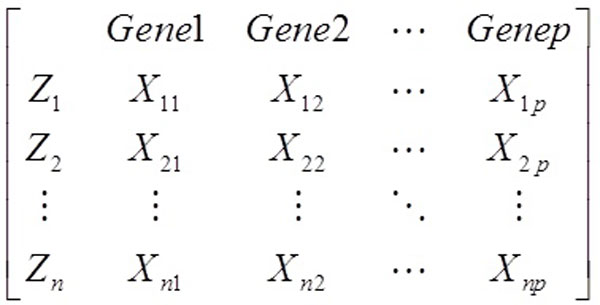
**Example of a gene expression matrix**.

In Figure 1, for each sample *Z*, we measure the expression levels of *p *genes, let $X_{\text{ij}}$ denotes the expression level of the *j*th gene in the *Z_i _*sample, where j = 1,...p. The x^th ^column vector of matrix represents the expression profile of the gene *X*. In order to define the biweight midcorrelation(bicor) [23] of two numeric vectors $x=\left(x_{1},...,x_{m}\right)$ and $y=\left(y_{1},...,y_{m}\right)$, we first defines $u_{i}$, $v_{i}$ with  = 1,...,m:

(1) $$u_{i}=\frac{x_{i}-med\left(x\right)}{9mad\left(x\right)}$$

(2) $$v_{i}=\frac{y_{i}-med\left(y\right)}{9mad\left(y\right)}$$

Where *med*(x) is the median of x, *mad*(x) is the median absolute deviation of x, *mad*(x) is the median of new numeric vector which each number is absolute difference between original vector value and *med*(x), this lead us to the definition of *mad*(x) and weight $w_{i}$ for $x_{i}$, which are,

(3) $$mad\left(x\right)=med\left(\left|x_{i}-med\left(X\right)\right|\right)$$

(4) $$w_{i}^{\left(x\right)}={\left(1-u_{i}^{2}\right)}^{2}I\left(1-\left|u_{i}\right|\right)$$

Where the indicator $I\left(1-\left|u_{i}\right|\right)$ takes 1 if $1-\left|u_{i}\right|>0$ and 0 otherwise. Thus, the weight $w_{i}^{\left(x\right)}$ is close to 1 if $x_{i}$ is close tomed(x), approaches 0 when $x_{i}$ differs from by nearly 9mad(x), and is 0 if $x_{i}$ differs from med(x) by more than 9mad(x). An analogous weight $w_{i}^{\left(x\right)}$ can be defined for$y_{i}$. Given the weights, we can define biweight midcorrelation of  and as:

(5) $$bicor\left(x,y\right)=\frac{\sum_{i=1}^{m}\left(x_{i}-med\left(x\right)\right)w_{i}^{\left(x\right)}\left(y_{i}-med\left(y\right)\right)w_{i}^{\left(y\right)}}{\sqrt{\sum_{j=1}^{m}{\left[\left(x_{j}-med\left(x\right)\right)w_{j}^{\left(x\right)}\right]}^{2}}\sqrt{\sum_{k=1}^{m}{\left[\left(y_{k}-med\left(y\right)\right)w_{k}^{\left(y\right)}\right]}^{2}}}$$

It should be noted that the equations of biweight midcorrelation does not invole an explicit identification of outliers, and all elements whose weight $w_{i}$ = 0 can be considered outliers. The user can also set up the maximum allowed proportion of outliers using the argument "maxPOutliers", the "max POutliers" is interpreted as the maximum proportion of low and high outliers separately. The value of bicor ranges from -1 to 1. Where -1 represents the maximum negative correlation and 1 represents the maximum positive correlation. Zero represents irrelevant.

### 'Half-thresholding' strategy in constructing gene coexpression networks

Gene expression data has the characteristic of small samples and large number of genes, and contains noise and unrelated genes. Therefore need to use the appropriate strategy to extract disease-related genes. There are currently two accepted strategies, namely hard-thresholding and soft-thresholding, for inferring gene coexpression network from original gene coexpression values. Those strategies can remove noise and irrelevant genes effectively. However, hard-thresholding ignores continuous nature of the coexpression information and encodes gene connections in a binary fashion, dichotomizing the continuous correlation values to be coexpression and non-coexpression. It is sensitive to the choice of the threshold and may be result in the loss of co-expression information.

The soft-thresholding keeps all possible coexpression relationships and uses the power *β *(i.e. soft-threshold) to emphasize the original high coexpression values and reduce the original low coexpression values simultaneously. Although soft-threshold overcomes the disadvantages of the hard-threshold, it keeps noisy variations and unrelated gene information in its calculation. These interference information lower the accuracy of gene differential coexpression analysis, especially when soft-threshold strategy uses a low value as the power*β*. During our gene differential coexpression analysis, pair of gene expression datasets under disease and normal conditions are transformed to a pair of coexpression matrix. We calculate bicor coefficients over all gene pairs in each dataset. We use ${\text{m}}_{ij}$ to denote bicor coefficient between gene and gene  under normal condition, and  to denote bicor coefficient under disease condition. The 'half-thresholding' strategy [17] keep coexpression value in both coexpression matrix if at least one of the two coexpression values exceeds the threshold. For example, we keep $m_{12}$ and $n_{12}$ if they both exceed threshold value 0.4. In this way, we ignore 'non-informative relationship' whose correlation values in both networks are below the threshold and filer the gene pair, but thoroughly examine the possibly meaningful coexpression changes of values remaining in the two coexpression matrix.

### The 'biweight midcorrelation and half-thresholding' method (BMHT)

In our method, for each dataset, we calculate the biweight midcorrelation coefficients between the expression profiles of all gene pairs in normal condition and disease condition separately. The biweight midcorrelation coefficients matrix represents the original correlation structure in each condition. After calculated biweight midcorrelation coefficients of all gene pairs, the two datasets are encoded into a pair correlations matrix over all gene pairs. We then filter out non-informative correlation pairs using the half-thresholding strategy. This results in a new coexpression networks of gene set.

In gene expression data, for gene, the biweight midcorrelation coefficients between it and its N neighbors in the filtered set can be calculated from two vectors, i.e., X=(x_i1_,x_i2_,...,x_in_) and Y=(y_i1_,y_i2_,...,y_in_) for the two conditions. We calculate the differential coexpression value of gene using the following equation.

(6) $$dc_{i}\left(BMHT\right)=\sqrt{\frac{{\left(x_{i1}-y_{i1}\right)}^{2}+{\left(x_{i2}-y_{i2}\right)}^{2}+...+{\left(x_{in}-y_{in}\right)}^{2}}{n}}$$

This calculates the average coexpression change between a gene and its informative coexpression genes. Then we can use the *dc *values to rank genes. Naturally, the question arises, i.e., whether our findings are artifacts of the high dimensionality and low sample of the data? To assess this question, we apply permutation test to evaluate the statistical significance of gene differential coexpression value. Under the null hypothesis, we assume that all genes are mutually independent in both conditions. During the permutation test, we firstly randomly permute the disease and normal conditions of the samples M times, then calculate new Biweight Midcorrelation coefficents using 'half-thresholding' strategy based on the new values, finally calculate the *dc *statistics. For gene set c, the permutation *p*-value is:

(7) $$\left\{\sum_{m=1}^{M}I\left[dc\left(p_{c}^{T_{1}^{m}},p_{c}^{T_{2}^{m}}\right)\geq dc(p_{c}^{T_{1}},p_{c}^{T_{2}}\right]\right\}/M$$

Here I(*) is an indicator function. If the absolute value of the dc of the permuted experimental matrices is larger than that of the original dc, I = 1. Otherwise, I = 0. The $T_{1}^{m}$ and $T_{2}^{m}$ denote samples derived from the *m*-th permuted dataset. An estimated FDR is obtained by converting the *p*-values to *q*-values using Benjamini-Hochberg method [29]. The *p*-value for each gene can then be calculated. In our study, we considered M = 1000. As this method is based on the Biweight Midcorrelation and Half-thresholding, it is denoted as BMHT in this paper.

### The maximum clique analysis

More and more researchers realized that gene module is high related with disease, but not individual gene. In gene expression network, gene is only related with other genes. Based on the characteristic of no self-loop, the graph of gene coexpression network is a simple undirected graph, and the diagonal elements of gene coexpression matrix are all 0. The gene coexpression matrix is a square and symmetric matrix whose rows and columns correspond to the genes and whose element A_ij _denotes the coexpression relationship between genes. The graph of maximum clique network is a complete graph that every pair of nodes is joined by edge, and the adjacency matrix elements of the complete graph are all 1 except the diagonal elements. For a simple undirected graph G containing N nodes, its adjacency matrix $A={\left(a_{ij}\right)}_{N\times N}$ contains only 1 and 0. It is a square and symmetric matrix obviously. $a_{ij}=1$ represents that gene i and j is coexpressed, $a_{ij}=0$ means that gene i and j is not connected.

We set two thresholds T_1 _for adjacency matrix A_1 _in normal condition and T_2 _for adjacency matric A_2 _in disease condition. A_1_(i,j) set to 1 if the value of A_1 _(i,j) greater than or equal to T_1_, otherwise, A_1_(i,j) set to 0. A_2_(i,j) set to 1 if value of A_2_(i,j) less than or equal to T_2_, otherwise, A_2_(i,j) set to 0.We integrated A_1 _and A_2 _into a matrix A after we had intersection the corresponding elements of A_1 _and A_2_. A (i,j) = 1 means coexpression value of gene i and gene j in A_1 _greater than or equal to T_1_, and coexpression value of gene i and j in A_2 _less than or equal to T_2_. Equation 6 summarized the process. We excavated cliques which have biological significance from A adjacency matrix to further investigate gene regulatory networks.

(8) $$\begin{array}{c} ifA_{1}\left(i,j\right)\geq T_{1},thenA_{1}\left(i,j\right)=1,\\ elseA_{1}\left(i,j\right)=0\\ ifA_{2}\left(i,j\right)\leq T_{2},thenA_{2}\left(i,j\right)=1,\\ elseA_{2}\left(i,j\right)=0\\ A\left(i,j\right)=A_{1}\left(i,j\right)\&A_{2}\left(i,j\right) \end{array}$$

## Results and discussion

### Experiment result on simulate datasets

In this experiment, we analyzed a pair of simulated datasets used in a published study [27], which were generated based on two yeast signaling networks using SynTReN [28]. The simulate datasets consists of 20 genes, 50 samples in normal and disease conditions. MBP1_SWI6, PHO2, CLB5, TRP4, CLB6, FLO1, FLO10 were identified as differential coexpression genes. We evaluated BMHT method in terms of its capability to discover the differential coexpression genes from the simulated datasets, and compared it with methods, i.e., 'Log Ratio of Connection'(LRC), 'Average Specific Connection'(ASC), and 'Weighted Gene Coexpression Network Analysis'(WGCNA). We adopted the signed version of WGCNA and set the parameter β = 12[22]. The results are listed in Table 1. From Table 1 it can be seen that, the BMHT method can detected all seven differential coexpression genes and ranked them at top, while the other three methods cannot detect them accurate. Bold shown genes refers to the seven differential coexpression genes in the simulate datasets. We arranged the gene in accordance with the BMHT value.

**Table 1:** The twenty yeast genes involved in simulated dataset pair and the ranking of them by DCEA methods, signed WGCNA, ASC, and LRC separately.

Gene	BMHT	Signed-WGCNA	ASC	LRC
**MBP1_SWI6**	1	7	1	8
**PHO2**	2	3	2	5
**CLB5**	3	14	3	18
**TRP4**	4	4	7	9
**CLB6**	5	16	4	19
**FLO1**	6	1	10	7
**FLO10**	7	2	6	3
CDC11	8	9	12	17
SWI4	9	5	5	16
ACE2	10	18	15	1
SWI4_SWI6	11	6	8	10
CDC10	12	10	13	12
ACT1	13	17	14	6
HTB1	14	8	11	15
LEU2	15	11	9	13
CTS1	16	12	17	14
SPT16	17	15	18	11
HO	18	13	16	2
CAF4	19	19	19	4
SNF6	20	20	20	20

Bold shown genes refers to the seven differential coexpression genes in the simulate datasets. We arranged the gene in accordance with the BMHT value.

### Analyzing a Type 2 Diabetes (T2D) in rats

In this section, we apply the BMHT method to a pair of type 2 diabetes(T2D) in rats datasets (dataset pair T), which has been published in study [22]. Dataset pair T from dataset GSE3068 of Gene Expression Omnibus (GEO) database, which had been preprocessed by Hui Yu et.al[22]. Dataset pair T includes 4765 genes in 10 disease samples and 10 normal samples. After applied BMHT method to dataset pair T, we obtained 334 differential coexpression genes of 4765 genes, *p*-values cut-off 0.05, FDR<0.6% (see Additional file 1). Based on the good performance of *p*-values of most genes, we selected 7% as the differential coexpression genes. The false discovery rate (FDR) is estimated from the p-value of biweight midcorrelation using Benjamini-Hochberg method [29]. In the differential coexpression genes, Rapgef4 [30] and Notch2 [31] are reported T2D-related genes. We listed all 20 differential coexpression genes with T2D relevance in table 2. Some reported relevance in table 2 are obtained from Kyoto Encyclopedia of Genes and Genomes (KEGG) database, it is a bioinformatics resource for linking genomes to life and the environment. Although the rest genes are not be previously reported to be related with T2D, they should also deserve more attention. It is helpful for researchers to excavate gene modules and disease genes, establish a disease-related gene clusters, and further explore the pathogenesis of the disease and the biological function of the related-gene.

**Table 2:** Differential coexpression genes with existing evidence of T2D-relevance.

Gene	BMHT value	Reported Relevance
Ucp2	0.7423	T2D-related
Rapgef4	0.7375	T2D-related
Nr5a1	0.7256	T2D-related
Inpp5d	0.7222	KEGG rno04910;T2D-related
Pparg	0.7068	T2D-related;T2D-associated
Igf1r	0.6885	KEGG rno04940
Tsc2	0.6706	KEGG rno04930
Jak3	0.6670	KEGG rno04940
Serpine1	0.6628	T2D-relaed
Lipe	0.6589	KEGGrno04910;T2D-related
C3	0.6581	T2D-related
Il6	0.6566	T2D-related
Foxo1	0.6550	KEGG rno04930
Flot2	0.6442	T2D-related
Prkab1	0.6432	KEGGrno04910;T2D-related
Pik3r1	0.6417	T2D-related
Gsk3a	0.6413	KEGG rno04930
Irf8	0.6391	KEGG rno04930
Tagln	0.6358	T2D-related
Slc2a1	0.6327	KEGG rno04930
Trf1	0.6324	KEGG rno04940
Cel	0.6322	T2D-related
Cckar	0.6254	T2D-related
Irs2	0.6220	KEGG rno04930
Notch2	0.6211	T2Dassociated;T2D-related

rno04940: type I diabetes mellitus; rno04930: type II diabetes mellitus; rno04910: insulin signaling pathway.

### The maximum clique analysis of real gene expression data

In applications, the node and edge sets of the graphs we need to consider are that we interested. In section 3.2, we selected 334 differential coexpression genes (DCGs) based on BMHT method. The type 2 diabetes data contains some previously reported TD2-related genes. DCGs and T2D-related genes in GSE3068 dataset form a total of 595 gene subset K. Gene subset K_1 _represents the gene expression value in normal condition and gene subset K_2 _represents the gene expression value in disease condition. We got two 595 × 595 symmetric bicor coefficient matrix K_1 _and K_2 _after we had computed bicor values of every pair gene of gene subset with half-thresholding strategy. K_1_(i,j) means bicor value of i gene and j gene in normal sample and K_2_(i,j) represents bicor value of i gene and j gene in disease sample. In this study, we searched gene modules which have high bicor value in normal samples and low bicor value in disease samples for exploring the impact of disease on the gene coexpression. We set two thresholds T_1 _= 0.76 for K_1 _and T_2 _= 0.2 for K_2_. K_1_(i,j) set to 1 if absolute value of K_1_(i,j) greater than or equal to T_1_, otherwise, K_1_(i,j) set to 0. K_2_(i,j) set to 1 if absolute value of K_2 _(i,j) less than or equal to T_2_, otherwise, K_2_(i,j) set to 0. We integrated K_1 _and K_2 _into a matrix K. The K(i,j) set to 1if the values of K_1_(i,j) and K_2_(i,j) both equal to 1, otherwise, K(i,j) is 0. K is a square and symmetric adjacency matrix with only two different class elements, i.e., 0 and 1.

**Figure 2:**
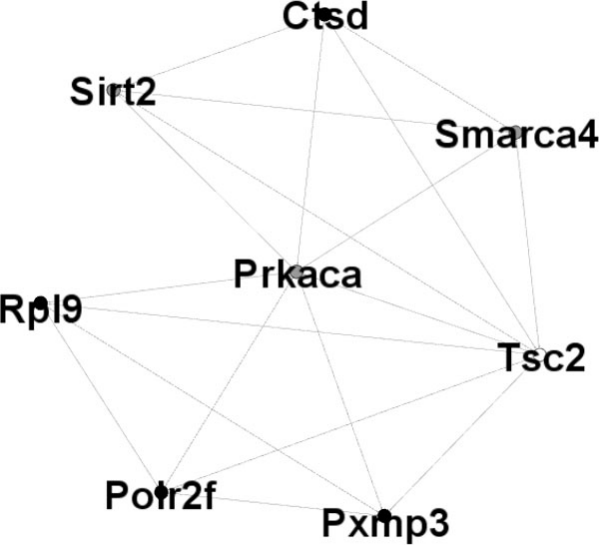
**Black spots refer to DCGs from GSE3068 dataset based on BMHT method**. Gray spots refer to T2D-related genes. White spot refer both DCG and T2D-related gene.

We analyzed the matrix as the adjacency matrix of graph. Each gene corresponds to one node of graph. K (i,j) = 1 also means node i and j node are connected by edge in correspond graph. We excavate cliques which have biological significance from the K adjacency matrix. and calculate the sum of each row or column of the K matrix. Which represents the number of edges that a gene connected to other genes. In order to improve the efficiency of search, we delete those isolated points whose numbers are 0 and set the minimum number of clique genes as 4. We mined 7 cliques all include 4 genes. The 7 cliques are combined into a gene module which includes 8 genes and 19 edges. The complete graph edge number of the gene function module is$c_{8}^{2}$, and the density is 0.68. We listed the genes of each clique in table 3. The result is shown in Figure 2. Bold shown genes refers to the four DCG selected in the GSE3068 dataset based on BMHT method. The other genes refer to DCG. Figure 2 is the gene module. Rpl9, Polr2f, Pxmp3 and Ctsd are DCGs. Smarca4, Sirt2 and Prkaca are T2D-related genes. Tsc2 is DCG and T2D-related gene.

**Table 3:** Genes of each clique.

Clique sequence number	Each Gene symbols of clique			
1	Tsc2	Smarca4	Sirt2	Prkaca
2	Tsc2	Smarca4	Sirt2	**Ctsd**
3	Sirt2	Tsc2	**Ctsd**	Prkaca
4	**Polr2f**	**Rpl9**	Prkaca	Tsc2
5	**Pxmp3**	**Rpl9**	Prkaca	Tsc2
6	**Pxmp3**	Tsc2	Prkaca	**Polr2f**
7	**Polr2f**	Prkaca	Smarca4	Tsc2

Bold shown genes refer to the four DCG selected genes in the GSE3068 dataset based on BMHT method. The other genes are DCG.

However, it is not easy to determine the optimal threshold for each specific study. Too large T_1 _or too small T_2 _will lead to small number edges and low density of the adjacency matrix K, which corresponding to a graph, and fail to find clique which meet the requirements. On the contrary, too small T_1 _or too large T_2 _will lead to overlapping cliques. These two cases have no sense for the analysis of biological process. So further investigation on optimizing thresholds procedure is necessary. In fact, the threshold can be determined based on the proportion of isolated points or density of the graph. The density is defined as the ratio of number of edges to the maximum number of edges. The maximum number of edges is the edge number of complete graph.

## Conclusion

In this paper, we proposed a new approach for differential coexpression analysis, which combine Biweight Midcorrelation and half-thresholding strategy and also applied maximum clique analysis to the specific gene set to further investigate gene regulatory networks. Biweight Midcorrelation is more robust for outliers and half-thresholding is an effective preprocess step of the proposed method. Experimental results on simulate datasets show that our method had better performance than three previsouly proposed methods. We also applied the proposed BMHT method to real dataset designed for T2D study, and 334 differential coexpression genes were selected, which may be a useful resource for T2D study and explore the biological function of the related-gene. In the future, we will focus on how to introduce new measure to scale the similarity of gene pairs.

## Competing interests

The authors declare that they have no competing interests.

## Supplementary Material

Additional file 1 **334 differental coexpression genes identified by our approach file format: **.doc. Click here for file

## Declarations section

Publication charges for this article have been funded by the National Science Foundation of China under Grant Nos. 61272339 & 61271098 & 61370109 and the Key Project of Anhui Educational Committee, under Grant No. KJ2012A005.

This article has been published as part of *BMC Bioinformatics *Volume 15 Supplement 15, 2014: Proceedings of the 2013 International Conference on Intelligent Computing (ICIC 2013). The full contents of the supplement are available online at http://www.biomedcentral.com/bmcbioinformatics/supplements/15/S15.
