# Supplementary material for: Gene differential coexpression analysis based on biweight correlation and maximum clique
Source: BMC Bioinformatics. 2014 Dec 3;15(Suppl 15):S3. doi: 10.1186/1471-2105-15-S15-S3 (PMC4271563; doi:10.1186/1471-2105-15-S15-S3)
Supplement: Additional file 1 — 334 differental coexpression genes identified by our approach file format: .doc. [file 1471-2105-15-S15-S3-S1.doc]

**Additional file 1** 334 DCGs identified by our approach

| Gene Id | Gene Symbol | Probeset | dc.value | Gene title |
| --- | --- | --- | --- | --- |
| 287177 | Ergic1 | rc_AA800719_at | 0.7855 | endoplasmic reticulum-golgi intermediate compartment (ERGIC) 1 |
| 84382 | Tcf4 | U09228_at | 0.7797 | transcription factor 4 |
| 50662 | Runx1 | L35271_at | 0.7650 | runt-related transcription factor 1 |
| 293186 | Lyve1 | rc_AI639246_at | 0.7556 | lymphatic vessel endothelial hyaluronan receptor 1 |
| 81809 | Tgfb2 | M96643_at | 0.7534 | transforming growth factor, beta 2 |
| 367586 | IgG-2a | L22654_at | 0.7532 | gamma-2a immunoglobulin heavy chain |
| 24450 | Hmgcs2 | M33648_at | 0.7511 | 3-hydroxy-3-methylglutaryl-Coenzyme A synthase 2 (mitochondrial) |
| 291885 | Mcm5 | rc_AA859768_at | 0.7510 | minichromosome maintenance complex component 5 |
| 641523 | LOC641523 | J00741_at | 0.7502 | immunoglobulin delta heavy chain constant region |
| 24906 | LOC24906 | AF041083_at | 0.7489 | RoBo-1 |
| 54315 | Ucp2 | AB010743_at | 0.7422 | uncoupling protein 2 (mitochondrial, proton carrier) |
| 25710 | Cd3d | X53430_at | 0.7413 | CD3 molecule delta polypeptide |
| 309621 | RT1-Ba | X07551cds_s_at | 0.7413 | RT1 class II, locus Ba |
| 307858 | Ldhd | rc_AI639504_at | 0.7409 | lactate dehydrogenase D |
| 252857 | Rapgef4 | U78517_at | 0.7374 | Rap guanine nucleotide exchange factor (GEF) 4 |
| 308069 | Slc12a7 | rc_AA799691_at | 0.7359 | solute carrier family 12 (potassium/chloride transporters), member 7 |
| 294269 | RT1-Da | M15562_at | 0.7355 | RT1 class II, locus Da |
| 499171 | Klf13 | rc_AA875534_at | 0.7354 | Kruppel-like factor 13 |
| 298426 | Nsun4 | rc_AA800200_at | 0.7346 | NOL1/NOP2/Sun domain family, member 4 |
| 362862 | Hsp90b1 | AA685903_at | 0.7325 | heat shock protein 90kDa beta (Grp94), member 1 |
| 50552 | Znf292 | L23077_at | 0.7316 | zinc finger protein 292 |
| 691001 | Ndufa3 | rc_AA875107_at | 0.7311 | NADH dehydrogenase (ubiquinone) 1 alpha subcomplex, 3 |
| 689046 | Ccdc84 | rc_AA800717_at | 0.7301 | coiled-coil domain containing 84 |
| 83781 | Lgals3 | J02962_at | 0.7281 | lectin, galactoside-binding, soluble, 3 |
| 170837 | Snrk | X89383_at | 0.7280 | SNF related kinase |
| 83826 | Nr5a1 | D42156cds_at | 0.7256 | nuclear receptor subfamily 5, group A, member 1 |
| 64522 | Slc5a2 | U29881_at | 0.7253 | solute carrier family 5 (sodium/glucose cotransporter), member 2 |
| 25394 | Bfsp1 | AB003104_at | 0.7224 | beaded filament structural protein 1 |
| 54259 | Inpp5d | U55192_at | 0.7221 | inositol polyphosphate-5-phosphatase D |
| 29257 | Rpl9 | X51706cds_at | 0.7217 | similar to ribosomal protein |
| 29185 | Cd37 | X53517_at | 0.7186 | CD37 molecule |
| 291133 | Aldh5a1 | L34821_at | 0.7176 | aldehyde dehydrogenase 5 family, member A1 |
| 300678 | Cd3g | S79711_at | 0.7175 | CD3 molecule, gamma polypeptide |
| 308565 | Klk8 | AJ005641_at | 0.7148 | kallikrein related-peptidase 8 |
| 171547 | Crispld2 | rc_AA859581_at | 0.7146 | cysteine-rich secretory protein LCCL domain containing 2 |
| 170568 | Dmbt1 | U32681_at | 0.7145 | deleted in malignant brain tumors 1 |
| 29268 | Tpsb2 | D38455_at | 0.7128 | tryptase beta 2 |
| 116689 | Ptpn6 | U77038_at | 0.7123 | protein tyrosine phosphatase, non-receptor type 6 |
| 25258 | Glycam1 | L08100_at | 0.7105 | glycosylation dependent cell adhesion molecule 1 |
| 25731 | Syt3 | D28512_at | 0.7102 | synaptotagmin III |
| 29687 | C1qb | X71127_at | 0.7100 | complement component 1, q subcomponent, B chain |
| 29252 | Gzmm | L05175_at | 0.7086 | granzyme M (lymphocyte met-ase 1) |
| 29227 | Nfib | AB012230_at | 0.7072 | nuclear factor I/B |
| 24646 | Abcb1b | M81855_at | 0.7069 | ATP-binding cassette, sub-family B (MDR/TAP), member 1B |
| 25664 | Pparg | AB011365_s_at | 0.7068 | peroxisome proliferator-activated receptor gamma |
| 83503 | Polr2f | AB017711_at | 0.7059 | polymerase (RNA) II (DNA directed) polypeptide F |
| 29715 | Slc8a1 | S67769_s_at | 0.7054 | solute carrier family 8 (sodium/calcium exchanger), member 1 |
| 64828 | B4galnt1 | D17809_at | 0.7051 | beta-1,4-N-acetyl-galactosaminyl transferase 1 |
| 25407 | Cd59 | rc_AA818025_at | 0.7050 | CD59 molecule, complement regulatory protein |
| 497874 | RGD1565784 | rc_AI639425_at | 0.7049 | RGD1565784 |
| 171123 | Rph3al | AF022774_at | 0.7046 | rabphilin 3A-like (without C2 domains) |
| 25454 | Gfra1 | U97142_at | 0.7033 | GDNF family receptor alpha 1 |
| 362795 | LOC362795 | X07189cds_at | 0.7028 | immunoglobulin G heavy chain |
| 29283 | Rpl29 | X68283_at | 0.7027 | ribosomal protein L29 |
| 25497 | Npap60 | U41845_at | 0.7025 | nuclear pore associated protein |
| 64664 | Arl3 | rc_AA799478_at | 0.7009 | ADP-ribosylation factor-like 3 |
| 25281 | Nup153 | rc_AA818240_at | 0.6979 | nucleoporin 153 |
| 29285 | Rps15 | E01534cds_s_at | 0.6979 | ribosomal protein S15 |
| 24797 | Sst | M25890_at | 0.6973 | somatostatin |
| 25556 | Il1rl1 | U04317_at | 0.6961 | interleukin 1 receptor-like 1 |
| 498600 | Fam32a | rc_AA892310_at | 0.6953 | family with sequence similarity 32, member A |
| 59074 | Stx8 | AF033109_at | 0.6951 | syntaxin 8 |
| 54311 | Timm17a | AB006450_at | 0.6948 | translocase of inner mitochondrial membrane 17 homolog A (yeast) |
| 315714 | Loxl1 | rc_AA800844_s_at | 0.6943 | lysyl oxidase-like 1 |
| 24162 | Acp2 | rc_AI234950_at | 0.6937 | acid phosphatase 2, lysosomal |
| 81759 | Renbp | D10233_at | 0.6934 | renin binding protein |
| 60460 | Hspa2 | X15705cds_at | 0.6933 | heat shock protein alpha 2 |
| 29619 | Btg2 | M60921_at | 0.6923 | BTG family, member 2 |
| 64356 | Nrgn | L09119_at | 0.6919 | neurogranin |
| 361215 | Auh | rc_AA859688_at | 0.6919 | AU RNA binding protein/enoyl-coenzyme A hydratase |
| 286924 | Gnat3 | X65747_at | 0.6902 | guanine nucleotide binding protein, alpha transducing 3 |
| 116639 | Cux1 | U09229_at | 0.6900 | cut-like homeobox 1 |
| 363210 | Phf3 | rc_AA894168_at | 0.6899 | PHD finger protein 3 |
| 50554 | Smad4 | rc_AI008639_at | 0.6891 | SMAD family member 4 |
| 29309 | Coq3 | L20427_at | 0.6887 | coenzyme Q3 homolog, methyltransferase (S. cerevisiae) |
| 64157 | Ddah1 | D86041_at | 0.6885 | dimethylarginine dimethylaminohydrolase 1 |
| 24718 | Reln | rc_AA893471_s_at | 0.6885 | reelin |
| 25718 | Igf1r | L29232_at | 0.6884 | insulin-like growth factor 1 receptor |
| 29681 | C1qbp | rc_AI178135_at | 0.6883 | complement component 1, q subcomponent binding protein |
| 501449 | Acat1 | D00512_at | 0.6882 | acetyl-coenzyme A acetyltransferase 1 |
| 29264 | Clip2 | AJ000485_at | 0.6878 | CAP-GLY domain containing linker protein 2 |
| 58949 | Ptafr | U04740_at | 0.6877 | platelet-activating factor receptor |
| 315463 | RGD1309188 | rc_AA800017_at | 0.6871 | similar to hypothetical protein BC011833 |
| 338401 | Crip2 | D17512_at | 0.6870 | cysteine-rich protein 2 |
| 29210 | Epha3 | U69278_at | 0.6869 | Eph receptor A3 |
| 84019 | Nae1 | U90829_at | 0.6851 | NEDD8 activating enzyme E1 subunit 1 |
| 83476 | Cyr61 | rc_AA800784_at | 0.6840 | cysteine-rich, angiogenic inducer, 61 |
| 171152 | Taf9b | U40188_at | 0.6838 | TAF9B RNA polymerase II, TATA box binding protein (TBP)-associated factor |
| 309255 | Mtmr10 | rc_AA894297_at | 0.6835 | myotubularin related protein 10 |
| 299511 | Rdh2 | U33500_at | 0.6832 | retinol dehydrogenase 2 |
| 116510 | Timp1 | rc_AI169327_at | 0.6829 | TIMP metallopeptidase inhibitor 1 |
| 117855 | Sf1 | AF079873_at | 0.6824 | splicing factor 1 |
| 64524 | Tkt | rc_AI059508_s_at | 0.6821 | transketolase |
| 54260 | Itpkb | X74227cds_at | 0.6820 | inositol 1,4,5-trisphosphate 3-kinase B |
| 25660 | Cd28 | X55288_at | 0.6818 | Cd28 molecule |
| 171116 | Opa1 | U93197_at | 0.6814 | optic atrophy 1 homolog (human) |
| 114495 | Map2k6 | rc_AI176689_at | 0.6796 | mitogen-activated protein kinase kinase 6 |
| 641625 | Slc41a3 | rc_AA799457_at | 0.6795 | solute carrier family 41, member 3 |
| 315697 | Imp3 | rc_AA799369_at | 0.6791 | IMP3, U3 small nucleolar ribonucleoprotein, homolog (yeast) |
| 81772 | Rps9 | X66370_at | 0.6782 | ribosomal protein S9 |
| 116591 | Fcgr2a | M32062_at | 0.6782 | Fc fragment |
| 29284 | Rps14 | rc_AA945806_at | 0.6782 | ribosomal protein S14 |
| 29464 | Slc6a6 | M96601_at | 0.6777 | solute carrier family 6 (neurotransmitter transporter, taurine), member 6 |
| 29143 | Rpl17 | X58389cds_s_at | 0.6775 | ribosomal protein L17 |
| 24818 | Tcp1 | rc_AA900850_at | 0.6773 | t-complex 1 |
| 29248 | Tnni3 | M92074_at | 0.6770 | troponin I type 3 (cardiac) |
| 94195 | S100a9 | L18948_at | 0.6769 | S100 calcium binding protein A9 |
| 288176 | RGD1309437 | rc_AA891759_at | 0.6766 | similar to RIKEN cDNA 2610528E23 |
| 64827 | Crisp1 | M31173mRNA#2_at | 0.6764 | cysteine-rich secretory protein 1 |
| 299154 | Churc1 | rc_AA859919_at | 0.6758 | churchill domain containing 1 |
| 313843 | Galm | rc_AA892666_at | 0.6753 | galactose mutarotase (aldose 1-epimerase) |
| 301552 | Mrpl44 | rc_H31711_at | 0.6747 | mitochondrial ribosomal protein L44 |
| 29543 | Timp2 | S72594_s_at | 0.6742 | TIMP metallopeptidase inhibitor 2 |
| 60331 | Atxn3 | Y12319cds_at | 0.6742 | ataxin 3 |
| 24914 | Lox | rc_AA875582_at | 0.6741 | lysyl oxidase |
| 81722 | Ager | L33413_at | 0.6738 | advanced glycosylation end product-specific receptor |
| 288516 | Eif3b | rc_AA875205_at | 0.6737 | eukaryotic translation initiation factor 3, subunit B |
| 24786 | Sod1 | M21060_s_at | 0.6737 | superoxide dismutase 1, soluble |
| 316005 | Arih2 | rc_AA799512_at | 0.6736 | ariadne homolog 2 (Drosophila) |
| 300726 | Etfa | rc_AA894174_at | 0.6735 | electron-transfer-flavoprotein, alpha polypeptide |
| 293711 | Hrasls5 | rc_AI638970_at | 0.6731 | HRAS-like suppressor family, member 5 |
| 24158 | Acadm | J02791_at | 0.6719 | acyl-Coenzyme A dehydrogenase, C-4 to C-12 straight chain |
| 64160 | Basp1 | D14441_at | 0.6718 | brain abundant, membrane attached signal protein 1 |
| 63868 | Hspd1 | rc_AA858640_s_at | 0.6718 | heat shock protein 1 (chaperonin) |
| 64515 | Cdc20 | AF052695_at | 0.6718 | cell division cycle 20 homolog (S. cerevisiae) |
| 687694 | RGD1566052 | rc_AA875129_at | 0.6715 | similar to elongation protein 4 homolog |
| 116669 | Vwf | U50044cds_at | 0.6706 | von Willebrand factor |
| 362588 | Ndufs5 | rc_AI009390_at | 0.6706 | NADH dehydrogenase (ubiquinone) Fe-S protein 5 |
| 24855 | Tsc2 | U24150_at | 0.6706 | tuberous sclerosis 2 |
| 25391 | Atp2a3 | M30581_at | 0.6701 | ATPase, Ca++ transporting, ubiquitous |
| 293152 | Art2b | M85193_at | 0.6700 | ADP-ribosyltransferase 2b |
| 116657 | Cplx2 | U35099_at | 0.6699 | complexin 2 |
| 25488 | Ndufa5 | D86215_at | 0.6698 | NADH dehydrogenase (ubiquinone) 1 alpha subcomplex 5 |
| 24189 | Aldoa | M12919mRNA#2_at | 0.6696 | aldolase A, fructose-bisphosphate |
| 294797 | Il7r | rc_AA800754_at | 0.6695 | interleukin 7 receptor |
| 58942 | Cacnb4 | L02315_at | 0.6695 | calcium channel, voltage-dependent, beta 4 subunit |
| 170577 | Mark3 | rc_AA893247_at | 0.6694 | MAP/microtubule affinity-regulating kinase 3 |
| 25109 | Cd1d1 | D26439_at | 0.6693 | CD1d1 molecule |
| 65204 | Cnn1 | D14437_s_at | 0.6689 | calponin 1, basic, smooth muscle |
| 29386 | Mecp2 | M94064_at | 0.6689 | methyl CpG binding protein 2 |
| 361074 | Slc25a30 | rc_AA892522_at | 0.6688 | solute carrier family 25, member 30 |
| 297695 | Wbp11 | rc_AA892364_at | 0.6687 | WW domain binding protein 11 |
| 117266 | Krt14l | D63774_at | 0.6684 | keratin 14-like |
| 25284 | Amacr | U89905_at | 0.6683 | alpha-methylacyl-CoA racemase |
| 81756 | Rab13 | M83678_at | 0.6679 | RAB13, member RAS oncogene family |
| 311547 | Foxs1 | rc_AA875405_at | 0.6677 | forkhead box S1 |
| 298201 | RGD1311249 | rc_AA894318_at | 0.6674 | similar to RIKEN cDNA B230312A22 |
| 287167 | LOC287167 | rc_AI178971_at | 0.6671 | globin, alpha |
| 157074 | Sdha | rc_AA800250_at | 0.6670 | succinate dehydrogenase complex, subunit A, flavoprotein (Fp) |
| 25326 | Jak3 | D28508_at | 0.6670 | Janus kinase 3 |
| 60335 | Tgm1 | M57263_at | 0.6669 | transglutaminase 1, K polypeptide |
| 64202 | Calr | D78308_at | 0.6667 | calreticulin |
| 29677 | Psmc3 | U77918_at | 0.6662 | proteasome (prosome, macropain) 26S subunit, ATPase 3 |
| 83624 | Ppig | AF043642_at | 0.6658 | peptidylprolyl isomerase G |
| 246060 | Cdkn1c | AF053094_at | 0.6652 | cyclin-dependent kinase inhibitor 1C |
| 500152 | Mmrn1 | rc_AI639113_at | 0.6651 | multimerin 1 |
| 300974 | Mrpl3 | rc_AA800272_at | 0.6651 | mitochondrial ribosomal protein L3 |
| 54231 | Car2 | U60578cds_s_at | 0.6649 | carbonic anhydrase II |
| 116640 | Tnc | U09361_s_at | 0.6646 | tenascin C |
| 114612 | Bat1 | M75168_at | 0.6640 | HLA-B associated transcript 1 |
| 25061 | Crabp1 | rc_AA875025_at | 0.6638 | cellular retinoic acid binding protein 1 |
| 25547 | St8sia3 | U55938_at | 0.6636 | ST8 alpha-N-acetyl-neuraminide alpha-2,8-sialyltransferase 3 |
| 25317 | Fgf1 | X14232_at | 0.6635 | fibroblast growth factor 1 |
| 29476 | Glg1 | rc_AI176461_s_at | 0.6635 | golgi apparatus protein 1 |
| 286905 | Cyp4f5 | U39207_at | 0.6631 | cytochrome P450, family 4, subfamily f, polypeptide 5 |
| 29558 | Fcgrt | rc_AI180013_at | 0.6630 | Fc fragment of IgG, receptor, transporter, alpha |
| 24617 | Serpine1 | M24067_at | 0.6627 | serine (or cysteine) peptidase inhibitor, clade E, member 1 |
| 29265 | Mcpt1 | AF063851_at | 0.6627 | mast cell protease 1 |
| 63864 | Hsd17b10 | rc_AA945583_at | 0.6621 | hydroxysteroid (17-beta) dehydrogenase 10 |
| 360502 | Itfg3 | rc_AA799854_at | 0.6621 | integrin alpha FG-GAP repeat containing 3 |
| 94268 | Efna1 | D38056_at | 0.6620 | ephrin A1 |
| 313050 | Lck | rc_AA800684_at | 0.6617 | lymphocyte-specific protein tyrosine kinase |
| 58917 | Hpx | M62642_at | 0.6616 | hemopexin |
| 54223 | Adcy4 | M80633_at | 0.6609 | adenylate cyclase 4 |
| 24837 | Tnnt2 | M80829_at | 0.6603 | troponin T type 2 (cardiac) |
| 80841 | Fabp7 | U02096_at | 0.6603 | fatty acid binding protein 7, brain |
| 64154 | Gosr2 | rc_AI175208_at | 0.6603 | golgi SNAP receptor complex member 2 |
| 25278 | Cox6a2 | rc_AI171644_s_at | 0.6602 | cytochrome c oxidase, subunit VIa, polypeptide 2 |
| 24770 | Ccl2 | X17053cds_s_at | 0.6600 | chemokine (C-C motif) ligand 2 |
| 29426 | Rps4x | rc_AA799501_at | 0.6595 | ribosomal protein S4, X-linked |
| 29563 | Crabp2 | U23407_at | 0.6589 | cellular retinoic acid binding protein 2 |
| 25330 | Lipe | U40001_at | 0.6589 | lipase, hormone sensitive |
| 29707 | Gabra5 | L08494cds_s_at | 0.6588 | gamma-aminobutyric acid (GABA) A receptor, alpha 5 |
| 29192 | Psen1 | D82363_s_at | 0.6587 | presenilin 1 |
| 79563 | Grpel1 | U62940_at | 0.6585 | GrpE-like 1, mitochondrial |
| 297597 | Ing4 | rc_AA892937_at | 0.6582 | inhibitor of growth family, member 4 |
| 24232 | C3 | M29866_s_at | 0.6581 | complement component 3 |
| 24694 | Pth | S80127_s_at | 0.6578 | parathyroid hormone |
| 246212 |  |  | 0.6574 |  |
| 313837 |  |  | 0.6572 |  |
| 361814 | Sfrs3 | rc_AI011706_at | 0.6571 | splicing factor, arginine/serine-rich 3 |
| 24717 | Rho | Z46957_at | 0.6569 | rhodopsin |
| 25279 | Cyp24a1 | L04619_s_at | 0.6566 | cytochrome P450, family 24, subfamily a, polypeptide 1 |
| 295703 | Serping1 | rc_AA800318_at | 0.6565 | serine (or cysteine) peptidase inhibitor, clade G, member 1 |
| 24498 | Il6 | M26744_at | 0.6565 | interleukin 6 |
| 84024 | Pon1 | rc_AA817964_s_at | 0.6563 | paraoxonase 1 |
| 54267 | Maf | rc_AI070994_s_at | 0.6560 | v-maf musculoaponeurotic fibrosarcoma oncogene homolog (avian) |
| 292866 | Klk1b21 | M27217_at | 0.6558 | kallikrein 1-related peptidase b21 |
| 50671 | Fasn | M76767_s_at | 0.6555 | fatty acid synthase |
| 362324 | Mios | rc_AA892645_at | 0.6554 | missing oocyte, meiosis regulator, homolog (Drosophila) |
| 300149 | Ncaph2 | rc_AA893946_at | 0.6553 | non-SMC condensin II complex, subunit H2 |
| 289211 | Fcgr2b | X73371_at | 0.6553 | Fc fragment of IgG, low affinity IIb, receptor (CD32) |
| 25181 | Bgn | rc_AA859830_s_at | 0.6552 | biglycan |
| 294274 | RT1-DMa | U31598_s_at | 0.6552 | RT1 class II, locus DMa |
| 81679 | Jup | U58858_at | 0.6551 | junction plakoglobin |
| 361285 | Mllt10 | rc_AA799796_at | 0.6551 | myeloid/lymphoid or mixed-lineage leukemia (trithorax homolog, Drosophila); translocated to, 10 |
| 84482 | Foxo1 | rc_AA893671_at | 0.6550 | forkhead box O1 |
| 500860 | Zfp706 | rc_AA955950_at | 0.6549 | zinc finger protein 706 |
| 24577 | Myc | Y00396mRNA_at | 0.6546 | myelocytomatosis oncogene |
| 296710 | Arpc5l | rc_H32977_at | 0.6545 | actin related protein 2/3 complex, subunit 5-like |
| 29534 | Pxmp3 | E03344cds_s_at | 0.6541 | peroxisomal membrane protein 3 |
| 64638 | Rpl28 | X52619_at | 0.6539 | ribosomal protein L28 |
| 25600 | Utrn | AB011666_s_at | 0.6538 | utrophin |
| 25462 | Hspe1 | rc_AI170613_at | 0.6537 | heat shock protein 1 (chaperonin 10) |
| 60577 | Slc15a2 | rc_AI232096_at | 0.6533 | solute carrier family 15 (H+/peptide transporter), member 2 |
| 24553 | Met | U65007_at | 0.6533 | met proto-oncogene |
| 296313 | Myl9 | S77900_at | 0.6532 | myosin, light chain 9, regulatory |
| 24796 | Spn | rc_AI045440_at | 0.6531 | sialophorin |
| 117548 | Kif1b | AF083331_at | 0.6529 | kinesin family member 1B |
| 302941 | Fam100a | rc_AA859585_at | 0.6523 | family with sequence similarity 100, member A |
| 293103 | LOC293103 | rc_AA891221_at | 0.6522 | similar to RIKEN cDNA 0610007P06 |
| 296706 | Lhx2 | L06804_at | 0.6520 | LIM homeobox 2 |
| 24368 | Fh1 | J04473_at | 0.6513 | fumarate hydratase 1 |
| 307649 | Ciapin1 | rc_AA800176_at | 0.6513 | cytokine induced apoptosis inhibitor 1 |
| 64701 | Rpn2 | X55298_at | 0.6510 | ribophorin II |
| 25268 | Proc | X64336_at | 0.6507 | protein C |
| 363872 | Znf498 | rc_AA891600_at | 0.6503 | zinc finger protein 498 |
| 81827 | Psmc5 | AB000491_at | 0.6501 | proteasome (prosome, macropain) 26S subunit, ATPase, 5 |
| 170714 | Dynlrb1 | AF073839_s_at | 0.6499 | dynein light chain roadblock-type 1 |
| 24412 | Grin2d | D13213_s_at | 0.6498 | glutamate receptor, ionotropic, N-methyl D-aspartate 2D |
| 59101 | Sc65 | X65454_at | 0.6495 | synaptonemal complex protein SC65 |
| 369016 | Myadm | rc_AA866276_at | 0.6494 | myeloid-associated differentiation marker |
| 171577 | Epcam | AJ001044cds_at | 0.6492 | epithelial cell adhesion molecule |
| 360420 | Rdh7 | U18762_at | 0.6486 | retinol dehydrogenase 7 |
| 500690 | Map3k9 | rc_AA892737_at | 0.6484 | mitogen-activated protein kinase kinase kinase 9 |
| 501828 | LOC681196 | rc_AA891475_at | 0.6484 | similar to paired immunoglobin-like |
| 171082 | Atp5g2 | D13124_s_at | 0.6482 | ATP synthase, H+ transporting, mitochondrial F0 complex, subunit C2 (subunit 9) |
| 94197 | Rab14 | M83680_at | 0.6473 | RAB14, member RAS oncogene family |
| 29134 | Axin2 | AF017757_at | 0.6471 | axin2 |
| 689938 | Ndufc1 | rc_AI104679_s_at | 0.6471 | NADH dehydrogenase (ubiquinone) 1, subcomplex unknown, 1 |
| 29237 | Penk | S49491_s_at | 0.6470 | proenkephalin |
| 313173 | Cntfr | S54212_at | 0.6469 | ciliary neurotrophic factor receptor |
| 24251 | Cd53 | M57276_at | 0.6468 | Cd53 molecule |
| 299075 | Trappc6b | rc_AA800622_at | 0.6467 | trafficking protein particle complex 6B |
| 29639 | Fxyd2 | X70062_at | 0.6466 | FXYD domain-containing ion transport regulator 2 |
| 303970 | Bbx | rc_AI638939_at | 0.6465 | bobby sox homolog (Drosophila) |
| 29155 | Capn3 | AF052540_s_at | 0.6462 | calpain 3 |
| 361848 | Ddx50 | rc_AA799576_at | 0.6461 | DEAD (Asp-Glu-Ala-Asp) box polypeptide 50 |
| 499443 | Lims1 | rc_AA799637_at | 0.6458 | LIM and senescent cell antigen-like domains 1 |
| 287645 | Snf8 | rc_AA891666_at | 0.6457 | SNF8, ESCRT-II complex subunit, homolog (S. cerevisiae) |
| 56611 | Anxa2 | L13039_s_at | 0.6457 | annexin A2 |
| 83782 | Nme2 | M91597_s_at | 0.6456 | non-metastatic cells 2, protein (NM23B) expressed in |
| 29460 | Vgf | M74223_at | 0.6456 | VGF nerve growth factor inducible |
| 286989 | Ugt2b7 | U27518_at | 0.6454 | UDP glucuronosyltransferase 2 family, polypeptide B7 |
| 89812 | Pip4k2b | AF033355_at | 0.6453 | phosphatidylinositol-5-phosphate 4-kinase, type II, beta |
| 286962 | Cox6c1 | M20183_at | 0.6452 | cytochrome c oxidase subunit VIc-1 |
| 64353 | Pdlim5 | U48247_at | 0.6452 | PDZ and LIM domain 5 |
| 25205 | Il6st | M92340_at | 0.6452 | interleukin 6 signal transducer |
| 288667 | RGD1310861 | rc_AA891537_at | 0.6449 | similar to RIKEN cDNA 1500011H22 |
| 298003 | Galt | L05541_at | 0.6449 | galactose-1-phosphate uridylyltransferase |
| 25596 | Rpn1 | X05300_at | 0.6448 | ribophorin I |
| 311403 | Mtfr1 | rc_AA892399_at | 0.6446 | mitochondrial fission regulator 1 |
| 311362 | Frmd5 | rc_H31625_at | 0.6445 | FERM domain containing 5 |
| 24551 | Mdh1 | AF093773_s_at | 0.6445 | malate dehydrogenase 1, NAD (soluble) |
| 25648 | Slc7a1 | L10152_s_at | 0.6444 | solute carrier family 7 (cationic amino acid transporter, y+ system), member 1 |
| 295091 | Vom2r46 | AF053990_at | 0.6444 | vomeronasal 2 receptor, 46 |
| 83764 | Flot2 | AF023302_s_at | 0.6442 | flotillin 2 |
| 362463 | Fgfr1op2 | rc_AI639475_at | 0.6439 | FGFR1 oncogene partner 2 |
| 25527 | Ptgis | U53855_at | 0.6438 | prostaglandin I2 (prostacyclin) synthase |
| 29263 | Acvr2a | S48190_at | 0.6437 | activin A receptor, type IIA |
| 303369 | Lig3 | rc_AA875471_at | 0.6434 | ligase III, DNA, ATP-dependent |
| 116722 | Psmd10 | AB022014_at | 0.6432 | proteasome (prosome, macropain) 26S subunit, non-ATPase, 10 |
| 83803 | Prkab1 | X95577_at | 0.6431 | protein kinase, AMP-activated, beta 1 non-catalytic subunit |
| 25318 | Fnta | M81225_at | 0.6431 | farnesyltransferase, CAAX box, alpha |
| 290641 | Rpl18a | rc_AA799899_i_at | 0.6431 | ribosomal protein L18A |
| 29479 | Odf2 | X95272_s_at | 0.6431 | outer dense fiber of sperm tails 2 |
| 25156 | Vav1 | U39476_at | 0.6430 | vav 1 guanine nucleotide exchange factor |
| 311299 | Aven | rc_AA866272_at | 0.6430 | apoptosis, caspase activation inhibitor |
| 293481 | Tufm | rc_AA866234_at | 0.6430 | Tu translation elongation factor, mitochondrial |
| 24616 | Pah | M12337_at | 0.6430 | phenylalanine hydroxylase |
| 289508 | Rchy1 | rc_AA800053_at | 0.6430 | ring finger and CHY zinc finger domain containing 1 |
| 24464 | Hp | K01933_at | 0.6428 | haptoglobin |
| 293701 | Esrra | rc_AA799412_at | 0.6424 | estrogen related receptor, alpha |
| 25327 | Kcnc1 | X62840mRNA_s_at | 0.6422 | potassium voltage gated channel, Shaw-related subfamily, member 1 |
| 25577 | Ywhaq | D17614_at | 0.6422 | tryptophan 5-monooxygenase activation protein, theta polypeptide |
| 25677 | Mak | M35862_at | 0.6421 | male germ cell-associated kinase |
| 360504 | Hba-a2 | X56325mRNA_s_at | 0.6418 | hemoglobin alpha, adult chain 2 |
| 25066 | PVR | L12025_at | 0.6417 | poliovirus receptor |
| 25513 | Pik3r1 | D64045_s_at | 0.6416 | phosphoinositide-3-kinase, regulatory subunit 1 (alpha) |
| 362650 | Fblim1 | rc_AA875261_at | 0.6416 | filamin binding LIM protein 1 |
| 54271 | Tpsab1 | U67910_at | 0.6415 | tryptase alpha/beta 1 |
| 50686 | Gsk3a | X53427_at | 0.6412 | glycogen synthase kinase 3 alpha |
| 171163 | Slc6a13 | M95762_at | 0.6409 | solute carrier family 6 (neurotransmitter transporter, GABA), member 13 |
| 81814 | Tmsb4x | M34043_at | 0.6408 | thymosin beta 4, X-linked |
| 362896 | Stat6 | AF055292mRNA_at | 0.6406 | signal transducer and activator of transcription 6 |
| 25507 | Pcsk6 | D31854_s_at | 0.6406 | proprotein convertase subtilisin/kexin type 6 |
| 25435 | Eef2k | rc_AI059692_s_at | 0.6400 | eukaryotic elongation factor-2 kinase |
| 300732 | Pstpip1 | rc_AA893267_at | 0.6399 | proline-serine-threonine phosphatase-interacting protein 1 |
| 286911 | Prss3 | M16624_at | 0.6399 | protease, serine, 3 |
| 171080 | Slc17a1 | rc_AA892920_at | 0.6397 | solute carrier family 17 (sodium phosphate), member 1 |
| 24781 | Slc4a3 | J05167_at | 0.6397 | solute carrier family 4 (anion exchanger), member 3 |
| 25338 | Ninj1 | U72660_at | 0.6396 | ninjurin 1 |
| 29270 | Spr | M36410_at | 0.6395 | sepiapterin reductase (7,8-dihydrobiopterin:NADP+ oxidoreductase) |
| 24191 | Aldoc | M63656_s_at | 0.6394 | aldolase C, fructose-bisphosphate |
| 65205 | Mmp16 | D63886_s_at | 0.6391 | matrix metallopeptidase 16 |
| 292060 | Irf8 | rc_AA892259_at | 0.6390 | interferon regulatory factor 8 |
| 29287 | Rps19 | X51707cds_s_at | 0.6388 | ribosomal protein S19 |
| 29165 | Gzmk | rc_AA800703_at | 0.6386 | granzyme K |
| 25029 | Calcrl | L27487_at | 0.6382 | calcitonin receptor-like |
| 116664 | Atp6v1f | U43175_at | 0.6382 | ATPase, H transporting, lysosomal V1 subunit F |
| 25437 | Emd | X98377_at | 0.6382 | emerin |
| 116665 | Rere | U44091_at | 0.6381 | arginine-glutamic acid dipeptide (RE) repeats |
| 305494 | Aebp1 | rc_AA799755_at | 0.6381 | AE binding protein 1 |
| 24408 | Grin1 | L08228exon#22_s_at | 0.6380 | glutamate receptor, ionotropic, N-methyl D-aspartate 1 |
| 94198 | Psmb1 | rc_AA849722_at | 0.6379 | proteasome (prosome, macropain) subunit, beta type 1 |
| 79128 | Dab2 | U95178_s_at | 0.6377 | disabled homolog 2 (Drosophila) |
| 290673 | Atp13a1 | rc_AA893621_at | 0.6376 | ATPase type 13A1 |
| 297339 | Tex261 | rc_AA892260_at | 0.6372 | testis expressed 261 |
| 305420 | Anapc4 | rc_AA892346_at | 0.6370 | anaphase promoting complex subunit 4 |
| 24583 | Myh3 | K03467_s_at | 0.6368 | myosin, heavy chain 3, skeletal muscle, embryonic |
| 64198 | Pmpcb | D13907_at | 0.6368 | peptidase (mitochondrial processing) beta |
| 81613 | Ceacam1 | J04963_at | 0.6366 | carcinoembryonic antigen-related cell adhesion molecule 1 (biliary glycoprotein) |
| 50577 | Galr1 | U30290_at | 0.6366 | galanin receptor 1 |
| 25304 | Comp | X72914_at | 0.6363 | cartilage oligomeric matrix protein |
| 24771 | Sdc4 | rc_AA800059_at | 0.6363 | syndecan 4 |
| 117099 | Bdh1 | rc_AA817846_at | 0.6362 | 3-hydroxybutyrate dehydrogenase, type 1 |
| 294254 | Hspa1b | AA848563_s_at | 0.6361 | heat shock 70kD protein 1B (mapped) |
| 81647 | Atf2 | M65148_s_at | 0.6361 | activating transcription factor 2 |
| 361818 | Rsph1 | rc_H33120_at | 0.6360 | radial spoke head 1 homolog (Chlamydomonas) |
| 25745 | Myh9 | U15764_s_at | 0.6358 | myosin, heavy chain 9, non-muscle |
| 25418 | Dpysl3 | U52104_at | 0.6357 | dihydropyrimidinase-like 3 |
| 25123 | Tagln | M83107_at | 0.6357 | transgelin |
| 60671 | Gulo | D14564cds_s_at | 0.6356 | gulonolactone (L-) oxidase |
| 25504 | Oxt | K01701_at | 0.6354 | oxytocin, prepropeptide |
